# Interaction potential of Carmegliptin with P-glycoprotein (Pgp) transporter in healthy volunteers

**DOI:** 10.3109/21556660.2014.900065

**Published:** 2014-03-03

**Authors:** Olaf Kuhlmann, David Carlile, Johannes Noe, Darren Bentley

**Affiliations:** 1 Pharmaceutical Sciences, Pharma Research and Early Development (pRED), F. Hoffmann-La Roche Ltd., BaselSwitzerland; 2 Roche Products Ltd., Welwyn Garden CityUK; 3 Oncology Biomarker Development (OBD), F. Hoffmann-La Roche Ltd., BaselSwitzerland

**Keywords:** Carmegliptin, Verapamil, Norverapamil, DPP-IV, Diabetes, Transporter, Pgp

## Abstract

**Objective:**

The primary objective of this study was to investigate the interaction potential of carmegliptin with P-glycoprotein transporter *in vitro* and *in vivo*. A secondary objective was to investigate the safety and tolerability of carmegliptin alone or co-administered with verapamil.

**Research design and methods:**

The inhibition potential of carmegliptin was tested *in vitro* and in a non-randomized open-label study in 16 healthy male volunteers. On day 1 a single dose of carmegliptin (150 mg) was given, followed by a single dose of verapamil (80 mg) on day 7, on day 10 a single dose of carmegliptin (150 mg) together with verapamil (80 mg t.i.d.), and verapamil (80 mg t.i.d.) on days 11–14. Finally, on day 15 a single dose of 150 mg carmegliptin together with 80 mg t.i.d. verapamil was administered. Pharmacokinetic and safety parameters were assessed.

**Results:**

Carmegliptin showed *in vitro* a low cell permeability and was a good substrate for human MDR1 cells. When carmegliptin was taken with verapamil, the mean exposure and *C*_max_ to carmegliptin increased by 29% and 53%, respectively. Increases in exposure were slightly greater on the sixth day of verapamil dosing than on the first day. Verapamil *C*_max_ was 17% lower on average when given with carmegliptin than when verapamil was taken alone, and similar trends were apparent in corresponding norverapamil pharmacokinetics. All reported adverse events (*n* = 28) were mild in intensity, and verapamil had no apparent effect on the pattern or incidence of events.

**Conclusions:**

*In vitro*, carmegliptin is a substrate but not an inhibitor of human Pgp. Consistently, the co-administration of carmegliptin with verapamil altered the pharmacokinetics of carmegliptin slightly and moderately increased the exposure. Peak exposure of verapamil and its metabolite norverapamil tended to be lower when co-administered with carmegliptin. The combination of carmegliptin and verapamil was generally well tolerated. Although the observed overall changes in pharmacokinetics were small and dose adjustments in clinics are currently not expected, co-administration of carmegliptin with Pgp inhibitors should be carefully monitored in future clinical trials.

## Introduction

Carmegliptin is an investigational oral anti-hyperglycemic drug for the treatment of Type 2 diabetes as a monotherapy or in combination with other oral anti-hyperglycemic medications^1^. Carmegliptin selectively inhibits dipeptidyl-peptidase IV (DPP-IV), an enzyme that rapidly inactivates two incretin hormones released during food ingestion; glucagon-like peptide-1 (GLP1) and glucose-dependent insulinotropic polypeptide (GIP). Both hormones are integral components involved in the physiological control of insulin release and, therefore, in the regulation of blood glucose homeostasis. Inhibition of DPP-IV prolongs the half-life of circulating active GLP1 and GIP and has the potential to prolong their anti-hyperglycemic actions. These include stimulation of glucose-dependent insulin secretion from the pancreatic *β*-cells, inhibition of glucagon secretion, stimulation of insulin biosynthesis, and peripheral insulin sensitization. In clinical practice, DPP-IV inhibitors reduce glycemia, sustain insulin levels, reduce glucagon levels, and, ultimately, lead to significant reductions in HbA1c (glycated hemoglobin) in patients with Type 2 diabetes. Furthermore, in contrast to other oral anti-hyperglycemic medications, DPP-IV inhibitors do not cause weight gain and have a minimal risk of hypoglycemia.

Carmegliptin is a highly selective and potent reversible competitive inhibitor of DPP-IV *in vitro*^1,2^. *In vivo*, carmegliptin improved glucose tolerance in various Type 2 diabetes animal models in a dose-dependent manner. Improvements in fasting and postprandial plasma glucose, insulin secretion, hepatic glucose production, whole body insulin sensitivity, postprandial GLP1 excursion, and plasma fructosamine were also observed as well as beneficial effects on body weight, lipid metabolism, and pancreatic *β*-cell protection^3,4^.

P-glycoprotein (Pgp) transporter is located in various anatomical sites, including the apical membrane of excretory cells in the human intestine, liver, and kidney, and is, therefore, involved in the absorption, distribution, and elimination of chemically diverse compounds. Since carmegliptin was shown to be a substrate of Pgp *in vitro*, concomitant use of carmegliptin with drugs that are inhibitors of Pgp could potentially affect the absorption, distribution, or elimination of carmegliptin. The present study was, therefore, conducted to determine whether the pharmacokinetics of carmegliptin are altered when carmegliptin is co-administered with a Pgp inhibitor *in vivo*.

The Pgp inhibitor chosen for the study was verapamil. Both verapamil and its major metabolite norverapamil have inhibitory effects on Pgp activity *in vitro*^5^, and verapamil treatment has been shown to alter the pharmacokinetics of Pgp substrates *in vivo*. Verapamil is an accepted model Pgp inhibitor for clinical studies in healthy volunteers^6^, and is also clinically relevant for a population of patients with Type 2 diabetes. Verapamil is a calcium ion influx inhibitor used widely in clinical practice for the treatment of angina, arrhythmia, and essential hypertension. It is, therefore, likely to be co-administered to patients with Type 2 diabetes where hypertension and cardiovascular disease are common co-morbidities.

## Subjects and methods

### Clinical study

This non-randomized, open-label, single-center, five-treatment, five-period, fixed sequence study was conducted in 16 healthy male volunteers aged 18–50 years, with a body mass index of 18–32 kg/m^2^ (report no. 1025423, data on file, L. Hoffmann–La Roche). Exclusion criteria included smoking (>5 cigarettes/day or equivalent), use of any medication containing herbal drugs, multivitamins (permitted 2 days before each dosing), and history or evidence of alcohol or other substance abuse or addiction.

The study was conducted at the Roche Center for Applied Clinical Development in Welwyn Garden City, UK, in full compliance with the principles of the Declaration of Helsinki (1996) and subsequent amendments and the Good Clinical Practice guidelines. Written informed consent was obtained from all participants before screening. The study protocol was approved by the Welwyn Clinical Pharmacology Ethics Committee, The University of Hertfordshire, Hatfield, UK.

Each subject received a single dose of carmegliptin alone, a single dose of verapamil alone, a single dose of carmegliptin on the first day of verapamil repeated dosing followed by verapamil dosing to steady state, then a single dose of carmegliptin in combination with steady state verapamil. Hence, the five treatments were:

- A single 150 mg dose of carmegliptin (Day 1, ‘Carmegliptin single dose’);
- A single 80 mg dose of verapamil (Day 7, ‘Verapamil single dose’);
- A single 150 mg dose of carmegliptin plus verapamil 80 mg t.i.d. (Day 10, ‘Carmegliptin + acute verapamil’);
- Verapamil 80 mg t.i.d. for 4 days (Days 11–14, ‘Verapamil t.i.d.’); and
- A single 150 mg dose of carmegliptin plus verapamil 80 mg t.i.d. (Day 15, ‘Carmegliptin + chronic verapamil’).

Screening procedures included a complete medical history and a physical examination between 2–28 days before the first dosing day. On the morning of day – 1, eligible subjects were admitted to the unit for baseline ECG, clinical laboratory and urine chemistry safety tests. Subjects started the fixed treatment sequence on day 1 following an overnight fast of at least 8 h.

Safety assessments during the treatment period included all adverse events, clinical laboratory safety tests (days 3, 9, and 14), urine chemistry (days 1, 10, and 15), 24-h ECGs (days 1, 10, and 15), and Lead-2 telemetry (days 1, 10, and 15). Subjects were resident in the unit on four occasions (day – 1 to day 3, day 6 to day 8 [morning], day 9 [evening] to day 12, and day 14 to day 17). Ambulatory visits were also required on day 8 (evening) and day 9 (morning) for pharmacokinetic sampling.

A follow-up medical examination was conducted within 8 days of the final study drug administration.

### Pharmacokinetic assessments

Blood and urine samples for the pharmacokinetic measurements of carmegliptin were collected on days 1, 10, and 15 (at pre-dose, 0.25, 0.5, 0.75, 1, 1.5, 2, 3, 4, 6, 8, 10, 12, 16, 24, 36, and 48 h post-dose). Blood samples for the pharmacokinetic measurements of verapamil and its major metabolite, norverapamil, were collected on days 7 (at pre-dose, 0.25, 0.5, 0.75, 1, 1.5, 2, 3, 4, 6, 8, 10, 12, 16, 24, 36, and 48 h post-dose), 10 and 15 (at pre-dose, 0.25, 0.5, 0.75, 1, 1.5, 2, 3, 4, 6, 8, 10, 12, 16, and 24 h post-dose). On days 1, 7, 10, and 15, subjects remained fasted until 4 h after dosing when they consumed a standardized meal.

For carmegliptin, 3 mL venous blood samples were taken at each time point into the appropriate blood collection tube with EDTA as anticoagulant. For verapamil and norverapamil, 6 mL venous blood samples were taken into the appropriate blood collection tube with lithium heparin as anticoagulant. In both cases, plasma was separated by centrifugation at 1500 g (4°C, 10 min) within 30 min of blood collection.

Urine samples for the pharmacokinetic measurements of carmegliptin were collected on days 1, 10, and 15 at 0–4 h, 4–12 h, 12–24 h, and 24–48 h post-dose. Subjects were requested to void their bladder within 30 min prior to carmegliptin dosing. All urine produced by subjects during each sampling interval was collected in pre-weighed polyethylene containers. The urine was refrigerated (2–8°C) between collections and, at the end of each collection interval, an aliquot of ∼10 mL was transferred to polyethylene tubes.

Plasma and urine samples were analyzed for carmegliptin by high-performance liquid chromatography combined with tandem mass spectrometric detection at F. Hoffmann-La Roche Ltd, Basel, Switzerland. The lower limit of quantification was 1 ng/mL for plasma and 20 ng/mL for urine. The precision for carmegliptin in plasma and urine quality control (QC) samples ranged from 3.1–11.6% and from 4.7–8.0%, respectively. The accuracy for carmegliptin in plasma and urine QC samples ranged from 98.5–103.0% and from 107.0–107.4%, respectively.

Plasma samples were analyzed for verapamil and norverapamil at AAIPharma Deutschland GmbH & Co. KG, 89231 Neu-Ulm, Germany. The lower limit of quantification was 2.50 ng/mL, with a calibration range up to 200 ng/mL. The precision (CV) of the assay was in the range of 2.0–3.1% for verapamil and 2.5–4.5% for norverapamil. The accuracy of the assay was in the range 98.7–99.8% for verapamil and 100.7–101.8% for norverapamil. Both bioanalytical methods (carmegliptin and verapamil) were fully validated.

For carmegliptin, the following pharmacokinetic parameters were estimated in plasma and urine samples collected after study drug dosing on days 1, 10, and 15: AUC_0–24_, AUC_last_, AUC_inf_, *C*_max_, *t*_max_, *t*1/2, CL/F, Vz/F, Ae0-48, Fe, and CLR.

For verapamil and norverapamil, AUC_0–6_, AUC_last_, AUC_inf_, *C*_max(6 h)_, and *t*_max_ were estimated on day 7 and AUC_0–6_, *C*_max(6 h)_, and *t*_max_ on days 10 and 15, respectively.

For these model-independent estimations, WinNonlin version 4.1 (Pharsight Corporation, Mountain View, CA) was used.

### Safety assessments

All adverse events (AEs) occurring during the clinical study were recorded and their intensity was assessed by investigators as mild, moderate, or severe. AEs occurring before first dosing were reported as baseline signs and symptoms. An AE was considered mild if it caused noticeable discomfort but did not disrupt normal daily activities, moderate if it caused discomfort sufficient to reduce or affect daily activities, and severe if it resulted in an inability to work or perform normal daily activities. Investigators also assessed each AE’s relationship to treatment as unrelated, remote, possible, or probable. Factors used to determine the relationship to treatment included the temporal association of the AE with administration of study drug, the known pattern of response to the suspected drug, and re-appearance of the AE on re-challenge with the drug.

Vital signs, systolic and diastolic blood pressure, heart rate, and oral temperature were assessed after the subject had been in a supine position for at least 5 min. Vital signs were recorded at the following time-points: Screening visit, treatment period (days 1, 10, and 15 pre-dose and at 2, 6, and 12 h post-dose, and pre-dose only on days 3, 7, and 11–14), and at the follow-up visit. In addition, 12 lead-ECGs were recorded at screening visit day – 1, on days 1, 10, and 15 (pre-dose and at 2, 6, and 12 h post-dose) and at the follow-up visit. Heart rate, QT, QTcB, PR, and QRS were captured directly from the ECG machine. QTcF and RR were derived. T-wave morphology, the occurrence of U-waves, and any other observed abnormalities were additionally recorded. On days 1, 10, and 15, subjects were monitored for 8 h following each dose of carmegliptin for overt signs of arrhythmias. The CRF captured whether the results were normal or abnormal.

### Statistical analysis

The planned sample size of 16 subjects was determined to ensure that there was 80% power for the 90% confidence interval (CI) for the relative effect of verapamil on carmegliptin exposure to not extend more than 1.5-times above or 1/1.5- (i.e., 0.667) times below the true relative effect. In order to be conservative and to ensure the sample size was large enough to allow a proper assessment of the effect of verapamil on carmegliptin, a coefficient of variation of 37.0% for the within-subject variability was assumed on the basis of earlier clinical studies with this compound. Carmegliptin, verapamil, and norverapamil plasma concentrations, together with their computed pharmacokinetic parameters, were summarized descriptively (mean, median, geometric mean, SD, coefficient of variation, minimum, maximum, and number of observations).

### *In vitro* transporter studies with carmegliptin

Wild type and transfected MDCKII (ATCC #CCL34, cocker spaniel kidney epithelial cell line) and the LLCPK (ATCC #CL-101, porcine kidney epithelial cell line) cell lines were obtained from Dr A. Schinkel (The Netherlands Cancer Institute, Amsterdam, The Netherlands) and used under license agreement. To test if carmegliptin is a substrate of P-glycoprotein (Pgp), mouse mdr1a or human MDR1 transfected MDCKII or LLCPK-cell lines (M-MDR1, L-MDR1, L-Mdr1a) were used. The non-transfected wild-type cell lines MDCKII or LLCPK were used as controls. The quality of cell monolayers was verified by lack of inulin leakage and the expression of Mdr1a/MDR1 was determined using model substrates digoxin (Mdr1a/MDR1). The inhibition potential of carmegliptin was tested using digoxin as a model substrate. Verapamil was used as an inhibitor. More assay details were recently published^7^.

## Results

### *In vitro* assessment

Carmegliptin is not an inhibitor of the adenosine triphosphate (ATP)-dependent efflux pump P-glycoprotein (Pgp; also known as ATP-binding cassette, sub-family B [MDA/TAP] member 1 [ABCB1]). However, *in vitro*, carmegliptin was shown to be a good substrate for rodent mdr1a and human MDR1. Intestinal secretion was saturable at high doses of carmegliptin (Table 1).

**Table 1.** *In vitro* P-glycoprotein transporter investigation.

		Cell lines used to study P-glycoprotein (transport ratios in apical direction)				
		Control cells		Cells expressing human protein		Cells expressing mouse protein
Compound	Inhibitor	MDCKII	LLCPK	M-MDR1	LMDR1		Lmdr1a
Digoxin (tracer)	–	5.3	1.4	10.7	14.3	11.8
	Verapamil (100 µM)	1.3	–	2.3	1.1	1.2
Carmegliptin (4 µM)	–	3.5–4.8	1.4	9.7	5.5	4.2
	Verapamil (100 µM)	1.2	–	1.8	0.8	0.9
Digoxin	–	–	–	16.0/18.7*	–	–
	Carmegliptin (30 µM)	–	–	28.7/22.3*	–	–

*Results of two independent experiments.

### Clinical study

Sixteen healthy male volunteers were enrolled in and completed the clinical study. A summary of their demographic characteristics is presented in Table 2.

**Table 2.** Summary of demographic characteristics of the healthy male volunteers (*n* = 16).

Parameter	Value
Race no. (%)	
White	13 (81)
Black	3 (19)
Age, years	
Mean (SD)	35.3 (11.1)
Range	18–48
Weight, kg	
Mean (SD)	84.5 (8.8)
Range	64.1–97.7
Height, cm	
Mean (SD)	180.4 (7.0)
Range	165–192
Body mass index, kg/m^2^	
Mean (SD)	26.0 (3.2)
Range	21.2–31.7

### Pharmacokinetic assessments

#### Carmegliptin with acute verapamil

**Figure 1.**
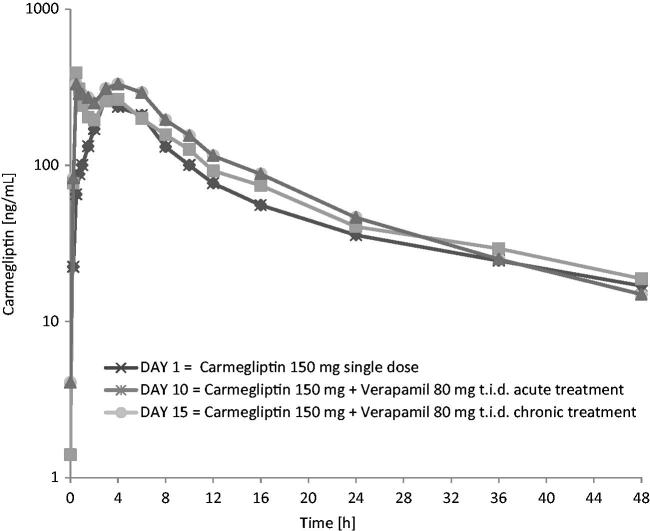
Carmegliptin: Mean plasma concentration vs time profiles on days 1, 10, and 15 (linear scale, 0–12 h).

**Figure 2.**
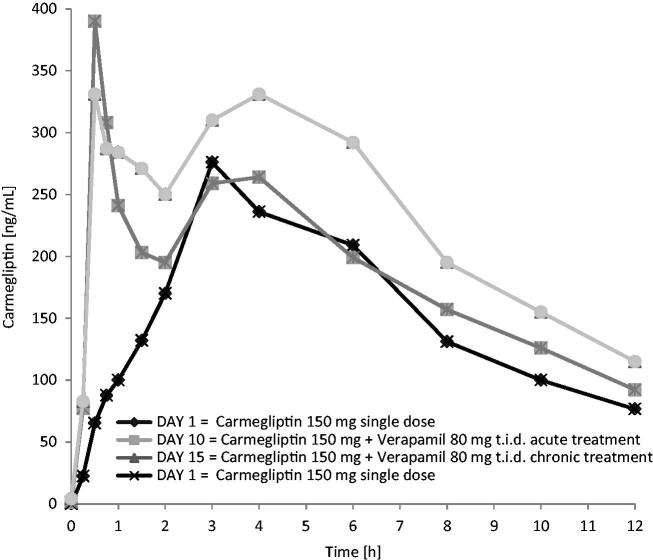
Carmegliptin: Mean plasma concentration vs time profiles on days 1, 10, and 15 (log-linear scale, 0–48 h).

**Figure 3.**
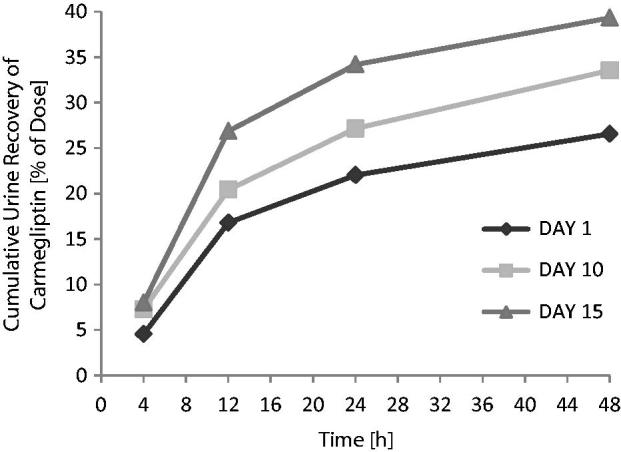
Carmegliptin: Mean cumulative urine recovery on days 1, 10, and 15.

When a single 150 mg dose of carmegliptin was taken on the first day of a verapamil 80 mg t.i.d. dosing regimen, the pharmacokinetics of carmegliptin was altered and exposure to carmegliptin was moderately increased. Plasma concentrations of carmegliptin increased rapidly post-dose, attaining maximum levels ∼2.25 h earlier (median *T*_max_ = 0.75 h) than when carmegliptin was administered alone (median *T*_max_ = 3.00 h) (Table 3). Two carmegliptin plasma concentration peaks were observed in most individuals, but, in contrast to observations when carmegliptin was administered alone, the earlier peak (∼1 h) tended to predominate (Figures 1 and 2). After the second peak, plasma concentrations of carmegliptin declined in a similar multi-phasic manner to that observed when carmegliptin was taken alone, and the estimated carmegliptin *t*_1/2_ was unchanged (Figures 1 and 2, Table 3). Peak plasma concentrations and total exposure to carmegliptin were both increased when carmegliptin was administered in combination with verapamil. Compared to values in the absence of verapamil (day 1), AUC_inf_ was 19% higher and *C*_max_ was 48% higher on average when carmegliptin was given with verapamil (Table 3). The changes in carmegliptin total exposure after administration with verapamil were reflected in changes in estimates of apparent oral clearance (CL/F) and volume of distribution (VzF). The fraction of the administered dose excreted unchanged in the urine (Fe) over 48 h was higher when carmegliptin was given with verapamil than when carmegliptin was taken alone (33 vs 26%), whereas estimated renal clearance (CL_r_) of carmegliptin was unchanged (13.6 vs 12.9 L/h) (Figure 3, Table 3). In both cases, the majority of the total eliminated in the urine was recovered within the first 24 h after dosing.

**Table 3.** Carmegliptin: Summary of pharmacokinetic parameters on days 1, 10, and 15.

	Geometric mean (CV%)		
	Carmegliptin single dose (day 1)	Carmegliptin + acute verapamil (day 10)	Carmegliptin + chronic verapamil (day 15)
*n*	16	16	16
AUC_0–24_ (h·ng/mL)	2410 (27.8)	2950 (25.5)	3620 (28.0)
AUC_last_ (h·ng/mL)	3010 (26.4)	3650 (24.1)	4280 (26.7)
AUC_inf_ (h·ng/mL)	3570 (23.9)	4250 (23.4)	4590 (26.3)
*C*_max_ (ng/mL)	342 (42.2)	505 (48.9)	522 (42.3)
*t*_max_ (h)*^a^*	3.00 (1.50–6.00)	0.75 (0.48–5.98)	1.50 (0.50–6.00)
*t*_1/2_ (h)	22.4 (18.9)	21.8 (18.8)	14.4 (12.4)
CL/F (L/h)	42.0 (23.9)	35.3 (23.4)	32.7 (26.3)
V_z_/F (L)	1360 (30.0)	1110 (30.0)	678 (30.0)
F_e_ (%)	26.0 (22.7)	33.1 (17.7)	38.7 (18.1)
CL_r_ (L/h)	12.9 (14.4)	13.6 (10.1)	13.6 (14.7)

*^a^*Median (range) for *t*_max_.

#### Carmegliptin with chronic verapamil

When a single 150 mg dose of carmegliptin was taken on the sixth day of a verapamil 80 mg t.i.d. dosing regimen, the pharmacokinetics of carmegliptin was again altered and exposure to carmegliptin was moderately increased.

Plasma concentrations of carmegliptin increased rapidly post-dose, attaining maximum levels ∼1.5 h earlier (median *T*_max_ = 1.5 h) than when carmegliptin was administered alone (Table 3). Again, two carmegliptin plasma concentration peaks were observed in most individuals, but, in contrast to observations when carmegliptin was administered alone, the earlier peak (∼1 h) tended to predominate (Figures 1 and 2). After the second peak, plasma concentrations of carmegliptin declined in a similar multi-phasic manner to that observed when carmegliptin was taken alone, although the rate of decline was faster and *t*_1/2_ was shorter (Figures 1 and 2, Table 3).

As seen with acute verapamil treatment, peak plasma concentrations and total exposure to carmegliptin were higher when carmegliptin was administered in combination with chronic verapamil than when carmegliptin was taken alone. AUC_inf_ was 29% higher and *C*_max_ was 53% higher on average compared to corresponding values when carmegliptin was administered alone (Table 3).

The changes in carmegliptin total exposure and half-life after administration with verapamil were reflected in changes in estimates of apparent oral clearance (CL/F) and apparent volume of distribution (V_z_/F).

The fraction of the administered carmegliptin dose excreted unchanged in the urine over 48 h was higher when carmegliptin was given with chronic verapamil than when carmegliptin was taken alone, whereas estimated renal clearance of carmegliptin was unchanged (Figure 3, Table 3).

#### Verapamil

**Figure 4.**
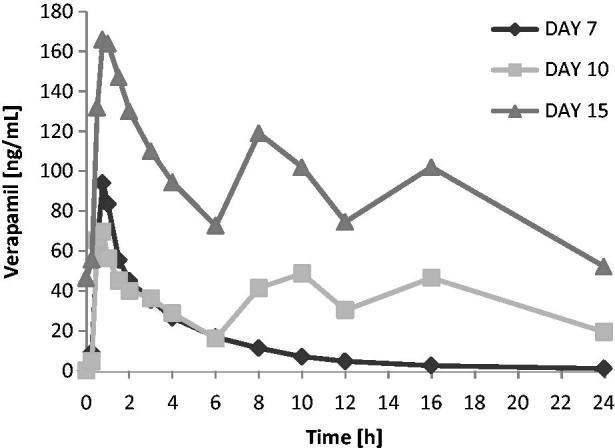
Verapamil: Mean plasma concentration vs time profiles on days 7, 10, and 15 (linear scale, 0–24 h).

Verapamil exposure over the 6 h following the first dose of the day tended to be lower when verapamil was administered in combination with carmegliptin. On average, verapamil *C*_max(6 h)_ and AUC_0–6_ were 17 and 11% lower, respectively, when a single dose of verapamil was administered with carmegliptin (day 10) than when verapamil was given alone (day 7) (Table 4). However, there was substantial inter-subject variability in verapamil exposure parameters, with CV% up to 92% and 61% for *C*_max(6 h)_ and AUC_0–6_, respectively (Table 4). As expected, verapamil exposure was higher at steady state on day 15 than after a single verapamil dose on day 7 (verapamil alone) or day 10 (verapamil in combination with a single dose of carmegliptin) (Figure 3). As steady state verapamil pharmacokinetics were not measured in the absence of carmegliptin, it was not possible to correctly assess the effect of carmegliptin on the steady state pharmacokinetics of verapamil. Therefore, comparisons of verapamil pharmacokinetic parameters data between days 7 and 10 are provided in Table 5 and Figure 4 only, and no conclusions can be made.

**Table 4.** Norverapamil: Summary of pharmacokinetic parameters on days 7 and 10.

	Geometric Mean (CV%)	
	Verapamil single dose (day 7)	Carmegliptin + acute verapamil (day 10)
*n*	16	16
AUC_0–6_ (h ng/mL)	203 (36.6)	185 (34.1)
*C*_max(6 h)_ (ng/mL)	63.8 (49.2)	51.2 (39.5)
*t*_max_ (h)*^a^*	1.00 (0.50–4.00)	0.99 (0.48–4.00)

*^a^*Median (range) for *t*_max_.

**Table 5.** Verapamil: Summary of pharmacokinetic parameters on days 7 and 10.

	Geometric mean (CV%)	
	Verapamil single dose (day 7)	Carmegliptin + acute verapamil (day 10)
*n*	16	16
AUC_0–6_ (h·ng/mL)	198 (60.5)	177 (59.3)
*C*_max(6 h)_ (ng/mL)	87.4 (92.3)	72.8 (83.7)
*t*_max_ (h)*^a^*	0.75 (0.50–3.00)	0.75 (0.48–3.02)

*^a^*Median (range) for *t*_max_.

#### Norverapamil

**Figure 5.**
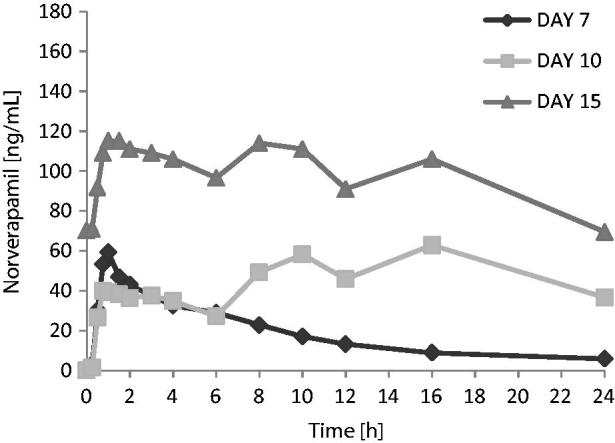
Norverapamil: Mean plasma concentration vs time profiles on days 7, 10, and 15 (linear scale, 0–24 h).

The apparent effect of carmegliptin on norverapamil was similar to the apparent effects of carmegliptin on parent verapamil. Norverapamil exposure over the 6 h following the first verapamil dose of the day appeared to be lower when a single dose of verapamil was administered with carmegliptin (day 10) than when verapamil was given alone (day 7). As expected, mean *C*_max(6 h)_ and AUC_0–6_ of norverapamil were higher at steady state on day 15 than after a single verapamil dose on day 7 or day 10 (Table 4 and Figure 5).

### Safety assessments

Carmegliptin was generally well tolerated, with only a few adverse events (AEs) during the study. Co-administration of carmegliptin with verapamil did not appear to have any effect on the safety profile of either compound. Between the time of first dosing and follow-up, 12/16 (75%) subjects reported a total of 28 AEs. The most frequently reported AEs were headache (8/16 subjects, 50%), catheter site hematoma (3/16 subjects, 19%), and pharyngolaryngeal pain (2/16 subjects, 13%). All other AEs were single occurrences. The adverse events, including headache, occurred sporadically throughout the fixed sequence treatment period in association with both carmegliptin and verapamil dosing.

The majority of AEs (22/28, 79%) were of mild intensity. AEs of moderate intensity were headache (two events), catheter site hematoma, pyrexia, back pain, and gastroenteritis. There were no events of severe intensity and no deaths.

Most AEs (20/28), including seven out of eight (88%) headaches, were considered by the investigator to be either remotely or possibly related to trial treatment (carmegliptin and/or verapamil). The remaining eight AEs (headache [1 event], catheter site hematoma, tooth ache, back pain, gastroenteritis, and joint injury) were considered to be unrelated to trial treatment.

## Discussion

*In vitro*, carmegliptin is a substrate but not an inhibitor of human Pgp. Therefore, mdr1/MDR1 could be involved in the intestinal secretion of carmegliptin. As a result, intestinal secretion was saturable at high doses of carmegliptin. It is likely that mdr1/MDR1 is also involved in biliary secretion and oral absorption of carmegliptin. The uptake process of carmegliptin by primary rat and human hepatocytes and in CHO cells was mainly passive diffusion (data not shown). Overall, based on these *in vitro* data, drug–drug interaction risk due to transporter is low due to multiple excretory routes, but this should be tested *in vivo*.

This study in healthy volunteers was, therefore, conducted to determine whether the pharmacokinetics of carmegliptin are altered when carmegliptin is co-administered with a Pgp inhibitor *in vivo*. Verapamil was chosen as a model Pgp inhibitor probe because it is clinically relevant for the intended target patient population and it does not present any major safety concerns for healthy volunteers.

Co-administration of verapamil with a single 150 mg dose of carmegliptin had modest effects on the pharmacokinetics of carmegliptin. The rate of carmegliptin absorption was faster and there was a clear alteration in the appearance of the plasma concentration v. time profiles. Two carmegliptin plasma concentration peaks were observed in most individuals, indicating two distinct absorption processes (‘fast’ and ‘slow’). While the ‘slow’ process appeared to predominate when carmegliptin was given alone, the ‘fast’ process tended to predominate when verapamil was co-administered. As a result, peak concentrations occurred 1.5–2.25 h earlier, which is consistent with inhibition of efflux and a ∼50% increased peak exposure on average. Total carmegliptin exposure was also higher (19–29% on average), although there was no apparent change in carmegliptin elimination half-life with acute verapamil treatment. Collectively, these data suggest that Pgp-mediated efflux limits the rate and extent of absorption of carmegliptin from the gastrointestinal tract under normal circumstances.

Although mean changes in carmegliptin exposure when verapamil was co-administered were modest, there was marked variability in individual responses. Individual subject *C*_max_ ratios between day 1 and day 10, for example, ranged from 0.50–3.42 (i.e., on the individual subject level, the change in *C*_max_ between day 1 and day 10 ranged from a decrease of 50% to an increase of 242%). Notably the magnitude of individual changes appeared to be inversely related to carmegliptin exposure in the absence of verapamil. Thus, subjects with the highest carmegliptin exposure when the drug was given alone had proportionally smaller increases in exposure when verapamil was co-administered. This suggests that absolute carmegliptin exposure from a specified dose in the presence of a Pgp inhibitor will effectively be constrained below a definable ceiling in all individuals.

Mean carmegliptin *t*_1/2_ was unchanged when carmegliptin was given at the start of verapamil treatment (21.8 vs 22.4 h), but was shorter when carmegliptin was given on the sixth day of verapamil dosing (14.4 vs 22.4 h). While the apparent change could be an artifact arising from failure to adequately characterize the terminal phase and accurately estimate *t*_1/2_ (i.e., sampling was only made up to 48 h post-dose and the kel is estimated from a period of only ∼1 × *t*_1/2_). However, this is considered unlikely because the effect is consistent across individuals and is clearly different between acute and chronic verapamil dosing. The most plausible mechanistic explanation is induction of Pgp expression by prolonged verapamil treatment. This would imply that the effects on carmegliptin exposure on the sixth day of verapamil dosing reflect a net balance between Pgp inhibition and induction, rather than being the consequence of only inhibition.

The fraction of the administered dose excreted unchanged in the urine over 48 h was higher when carmegliptin was given with verapamil (33.1 and 38.7%) than when carmegliptin was taken alone (26.0%). These increases in cumulative urinary recovery were proportionally similar to the effects on total exposure. In contrast, estimated renal clearance of carmegliptin was unaffected by concomitant verapamil treatment. Together, these data suggest that the increase in carmegliptin exposure and reduction in half-life from verapamil treatment are not caused by alterations in renal elimination, but rather are the result of changes in absorption or non-renal elimination processes. As carmegliptin half-life appears to be unaffected by acute verapamil dosing and there is a clear increase in the rate of drug absorption and a greater effect on peak exposure than on total exposure, it is suggested that verapamil treatment principally impacts carmegliptin absorption.

### Effect of carmegliptin on the pharmacokinetics of verapamil and norverapamil

A secondary objective of the study was to assess the effects of a single dose of carmegliptin on the pharmacokinetics of verapamil and norverapamil.

When a single dose of verapamil was administered in combination with a single dose of carmegliptin, mean *C*_max(6 h)_ and AUC_0–6_ of verapamil and its metabolite norverapamil tended to be lower than when a single dose of verapamil was administered alone (reductions of 17 and 11%, respectively). While the 90% CIs from ANOVAs of the verapamil data encompassed 100%, the lower 90% CI limits were below the accepted boundary for absence of an interaction (80%). However, since the study was not powered to demonstrate equivalence in exposure given the observed inter-individual variability in verapamil parameters (e.g., AUC_0–6_ CV%: 59–61%, *C*_max(6 h)_ CV%: 84–92%), it is not possible to definitively conclude whether carmegliptin has an effect on verapamil single dose pharmacokinetics. While the results appear to suggest that carmegliptin co-administration may decrease total verapamil exposure, the parameter AUC_0–6_ does not fully capture total exposure from a single dose and, in the absence of an appropriate measure such as AUC_inf_, no firm conclusions can be drawn about the effect of carmegliptin on verapamil bioavailability.

Verapamil exposure was higher after chronic dosing on day 15 than after a single dose on day 7 or acute dosing on day 10. This is expected because verapamil is likely to have reached steady state on day 15, and the bioavailability of verapamil is known to increase by a factor of 1.5–2 under a repeated dosing regimen^8^. However, as steady state verapamil pharmacokinetics were not measured in the absence of carmegliptin, it was not possible to correctly assess the effect of carmegliptin on the steady state pharmacokinetics of verapamil. Therefore, the protocol-specified comparisons of verapamil pharmacokinetics between a single dose or the first day of repeated dosing and steady state (i.e., day 15 vs day 7 and day 15 vs day 10) are not presented and no conclusions can be drawn about the effect of carmegliptin on steady state verapamil pharmacokinetics.

### Safety data

There were few adverse events (AEs) during the study, with headache, catheter site hematoma, and pharyngolaryngeal pain being the only AEs reported by more than one subject. The AEs occurred sporadically throughout the fixed sequence treatment period without clear association with either carmegliptin, verapamil, or combination treatment.

Although there were few occurrences of out-of-reference-range vital signs during the study, small but consistent decreases in mean blood pressure were apparent during the period of repeated dosing with verapamil (i.e., days 10–15). This is consistent with the known anti-hypertensive activity of verapamil^8^, and there is no evidence that combination with carmegliptin alters the pharmacodynamic effects of verapamil. Similarly, prolonged PQ(PR) intervals (>200 ms) were observed after drug administration in 7/16 (44%) subjects after carmegliptin plus verapamil combination treatment (days 10 and/or 15), but this occurred in only one subject after carmegliptin alone (day 1). However, three subjects also had at least one prolonged value before the start of study drug dosing. Furthermore, although ECGs were not collected after verapamil-only dosing (i.e., day 7), verapamil is known to alter the PQ(PR) interval^9^. While the possibility of additive or synergistic effects from combination treatment cannot be formally excluded, it is most likely that the effects on the PQ(PR) interval after carmegliptin plus verapamil treatment are attributable to verapamil. Apparent increases in heart rate/pulse rate occurred in all treatment periods, and appeared to reflect diurnal changes rather than specific treatment effects. It is, therefore, concluded that co-administration of carmegliptin with verapamil had no apparent effect on the safety profile of either compound.

There were few AEs during the study, and co-administration of carmegliptin with verapamil had no apparent effect on the pattern or incidence of AEs. There were no discontinuations, dose reductions, or serious AEs. No changes in vital signs, ECGs, or laboratory test variables were attributed to carmegliptin treatment.

## Conclusions

When a single 150 mg dose of carmegliptin was taken on the first or sixth day of a verapamil 80 mg t.i.d. dosing regimen, the pharmacokinetics of carmegliptin was altered and exposure to carmegliptin was moderately increased. Overall exposure to carmegliptin increased by up to 29% on average, and peak concentrations of carmegliptin increased by up to 53% on average. Increases in exposure were slightly greater on the sixth day of verapamil dosing than on the first day of verapamil dosing. Peak exposures of verapamil and its metabolite norverapamil tended to be lower when a single 150 mg dose of carmegliptin was co-administered with a single 80 mg dose of verapamil. No conclusions could be drawn about the effect of carmegliptin on the steady state pharmacokinetics of verapamil or norverapamil. The combination of carmegliptin and verapamil was generally well tolerated and had no apparent effect on the safety profile of either compound in healthy volunteers.

## Transparency

### Declaration of financial/other relationships

The authors are all employees of F. Hoffmann–La Roche Ltd. JDA Peer Reviewers on this manuscript have no relevant financial relationships to disclose.
